# Supplementary material for: The interactome of intact mitochondria by cross-linking mass spectrometry provides evidence for coexisting respiratory supercomplexes
Source: Mol Cell Proteomics. 2017 Dec 8;17(2):216–32. doi: 10.1074/mcp.RA117.000470 (PMC5795388; doi:10.1074/mcp.RA117.000470)
Supplement: Supplemental Data [file supp_RA117.000470_133922_0_supp_23978_fzffwf.zip › spectra_annotation/mito_spectra_annotation/100-1-1-1-7-1.pdf]

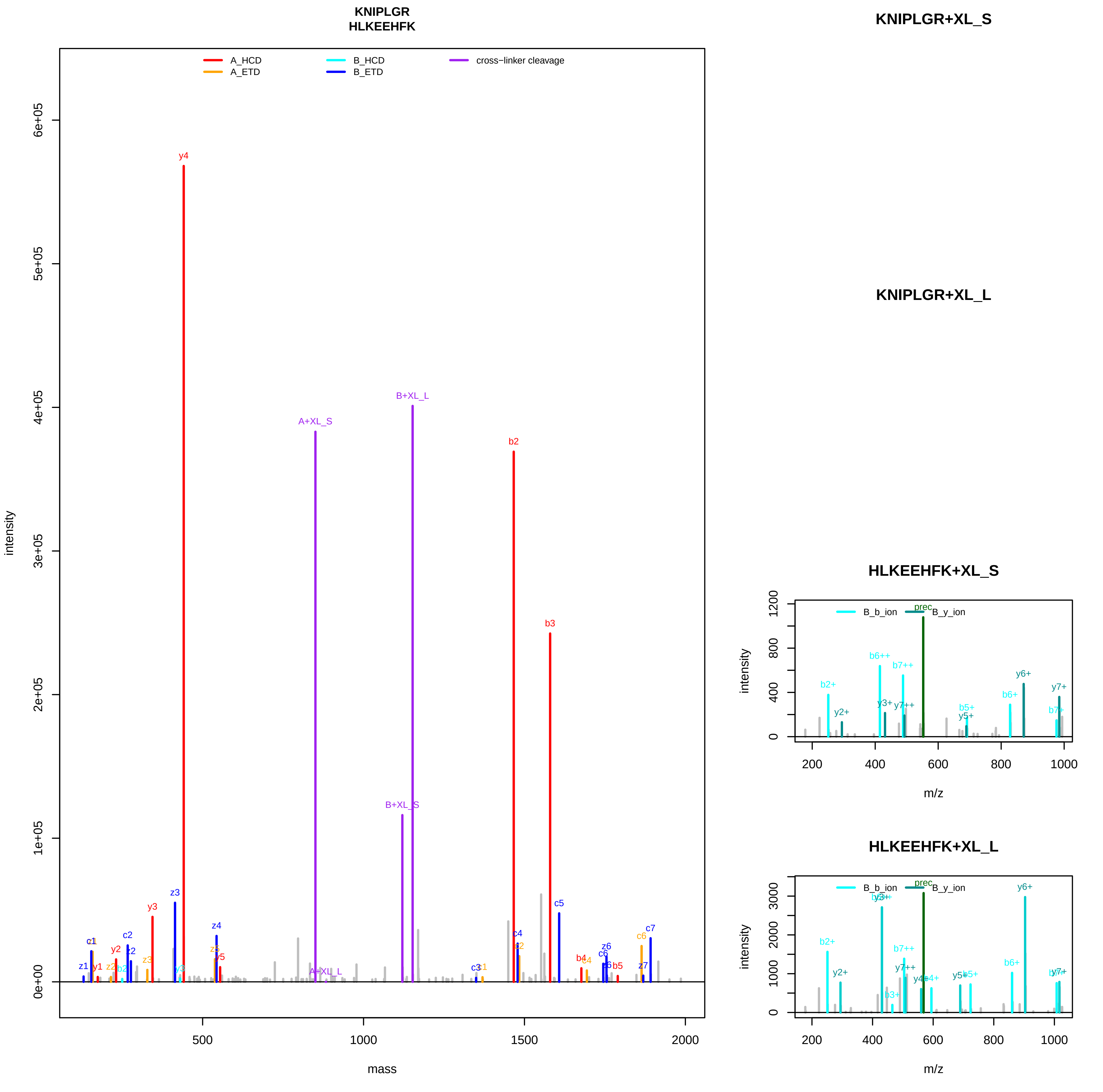
KNIPLGR
HLKEEHFK
A_HCD
A_ETD
B_HCD
B_ETD
cross−linker cleavage
intensity
mass
y4
A+XL_S
B+XL_L
B+XL_S
A+XL_L
b2
b3
y3
z3
z4
c5
c7
c6
c4
c3
c1
c2
z1
z2
y1
y2
z5
y5
b4
b5
z6
c6
z7
0e+00
1e+05
2e+05
3e+05
4e+05
5e+05
6e+05
500
1000
1500
2000
KNIPLGR+XL_S
KNIPLGR+XL_L
HLKEEHFK+XL_S
B_b_ion
B_y_ion
prec
b2+
y2+
b6++
b7++
y3+
y7++
b5+
y5+
b6+
y6+
y7+
b7+
m/z
HLKEEHFK+XL_L
b3+
y4+
b4+
b5+
